# Deep Learning Prediction of Retinal Thickness from Near-Infrared Fundus Photography: Toward Decentralized Quantitative Assessment of Diabetic Macular Edema

**DOI:** 10.3390/jpm16070361

**Published:** 2026-07-02

**Authors:** Behrouz Ebrahimi, Albert K. Dadzie, Mansour Abtahi, Masrur A. Sadhin, Daniel Kim, Srishti Kolla, Baoxin Li, R. V. Paul Chan, Michael J. Heiferman, Xincheng Yao

**Affiliations:** 1 Department of Biomedical Engineering, University of Illinois Chicago, Chicago, IL 60607, USA; bebrah2@uic.edu (B.E.); adadzi2@uic.edu (A.K.D.); abtahi@uic.edu (M.A.); msadh@uic.edu (M.A.S.); 2 Department of Computer Science, University of Illinois Chicago, Chicago, IL 60607, USA; dkim344@uic.edu (D.K.); baoxinli@uic.edu (B.L.); 3 Department of Ophthalmology and Visual Sciences, University of Illinois Chicago, Chicago, IL 60612, USA; skoll29@uic.edu (S.K.); rvpchan@uic.edu (R.V.P.C.); mheif@uic.edu (M.J.H.)

**Keywords:** diabetic macular edema, retinal thickness, near-infrared fundus imaging, deep learning, optical coherence tomography, decentralized screening, disease monitoring, treatment assessment, telemedicine

## Abstract

**Objective:** To predict pixel-wise retinal thickness maps from near-infrared (NIR) fundus images using deep learning (DL), and to identify image features in NIR fundus photographs serving as surrogate markers of retinal thickness, with implications for decentralized diabetic macular edema (DME) screening, progression monitoring, and treatment assessment. **Methods:** A DL model based on a U-Net architecture was trained on paired NIR fundus and OCT images from 531 eyes across three groups: healthy controls, diabetic retinopathy (DR) without DME, and DME. Model performance was evaluated using mean absolute error (MAE), root mean squared error (RMSE), structural similarity index (SSIM), and center-involved DME (ci-DME) classification at a central subfield thickness threshold of 300 µm. Controlled image manipulation experiments, including spatial disruption of vascular patterns, relocation of hard exudates, and contrast enhancement, were performed to identify image-level features serving as surrogate markers of retinal thickness. **Results:** The model achieved an MAE of 30.41 ± 18.68 µm, RMSE of 36.14 ± 21.05 µm, and SSIM of 0.87 ± 0.04 across the macula, with consistent performance across ETDRS subfields. For ci-DME classification, it achieved an accuracy of 84.1%, sensitivity of 69.1%, and specificity of 88.7%. Interpretability analyses were performed as qualitative assessments to visualize image regions contributing to model predictions. These analyses highlighted retinal vascular structures, hard exudates, and local contrast variations as visual features observed in relation to model outputs. **Conclusions:** NIR fundus images contain sufficient structural information to support pixel-wise retinal thickness estimation, with vascular architecture, hard exudates, and local contrast variations identified as image features potentially associated with model predictions. These findings suggest that NIR-based deep learning approaches may have potential applications in the assessment of diabetic macular edema and warrant further prospective and external validation to determine their role in screening, triage support, longitudinal monitoring, and treatment-related assessment, particularly in decentralized and re-source-limited care environments.

## 1. Introduction

Diabetic retinopathy (DR) is one of the leading causes of blindness and visual impairment worldwide [1]. With the global diabetic population projected to grow substantially over the coming decades [2], approximately one-third of individuals with diabetes are expected to develop DR [3,4]. Among its complications, diabetic macular edema (DME) is the most frequent vision-threatening manifestation at any stage of DR [5,6,7]. Timely identification and intervention are critical to prevent irreversible visual deterioration. Anti-vascular endothelial growth factor (anti-VEGF) therapy is the first-line treatment for DME [8,9,10], and eligibility is primarily determined by the presence of center-involved DME (ci-DME), defined by macular thickening on optical coherence tomography (OCT) [11,12]. Given the clinical risk associated with ci-DME, early and accurate identification is essential for guiding timely treatment decisions [13].

OCT is the clinical standard for DME diagnosis due to its ability to provide depth-resolved imaging of intraretinal and subretinal fluid [14,15,16]. However, OCT systems are expensive, require skilled operation, and are largely confined to specialist ophthalmic settings, limiting their deployment in primary care and telemedicine settings. In contrast, fundus photography is widely deployed in community screening programs and has served as the basis for AI-driven diabetic retinopathy detection tools such as EyeArt [17,18,19] and IDx-DR [20,21,22]. Nevertheless, conventional fundus photography lacks the depth-resolved structural information required for reliable DME diagnosis [23,24].

Deep learning (DL) has been explored for binary or graded classification of DME from fundus photographs, and for predicting scalar anatomical biomarkers such as central subfield thickness [25,26,27]. A smaller number of studies have explored pixel-level thickness map prediction from non-OCT modalities, using inputs such as fluorescein angiography or color fundus photography [28,29]. What remains unexplored is whether NIR reflectance imaging, with its nonmydriatic acquisition and depth-penetrating wavelength, can serve as a more physically motivated and clinically practical input for this task.

NIR imaging is inherently nonmydriatic: because longer-wavelength NIR light elicits minimal pupillary constriction, retinal imaging can be performed without pharmacological dilation in most cases. This substantially reduces examination time, eliminates dilation-associated patient discomfort, and removes a key barrier to eye care delivery in primary care and remote settings. Moreover, NIR light penetrates more deeply into ocular tissues than shorter visible wavelengths, reaching the retinal pigment epithelium (RPE) and the choroid [30,31]. As NIR light traverses and scatters over the full depth of the retina, the resulting two-dimensional fundus image encodes integrated optical signals from multiple retinal layers simultaneously. Consequently, structural changes associated with DME, such as intraretinal fluid accumulation, which alters tissue scattering and reflectance properties at depth, may produce measurable contrast signatures in the NIR fundus image. This provides a physical basis for the hypothesis that NIR fundus images carry latent retinal thickness information and motivates the application of DL to decode that information.

In this study, we demonstrate the prediction of full-field, pixel-wise retinal thickness maps from NIR fundus images using a DL model. A co-equal aim is to identify which image-level features in NIR fundus photographs serve as potential markers of retinal thickness, a question fundamental to understanding whether and why this prediction task is physically and informationally tractable. Through input manipulation experiments, we examine the contribution of vascular spatial patterns, hard exudates, and local contrast variations to the model’s predictions, and provide qualitative interpretability analyses of model behavior in relation to the optical properties of NIR imaging. The primary output of the model is spatially continuous retinal thickness estimation, from which ci-DME classification can be derived, enabling both qualitative interpretation and quantitative regional assessment. This feasibility study demonstrates the potential of combining nonmydriatic NIR fundus imaging with deep learning for retinal thickness estimation. This approach offers a cost-effective framework with potential implications for DME triage, referral reduction, longitudinal monitoring, and treatment response assessment in decentralized eye care settings.

## 2. Materials and Methods

### 2.1. Study Population and Preprocessing

This retrospective study was conducted in accordance with the ethical principles outlined in the Declaration of Helsinki and received approval from the Institutional Review Board (IRB) of the University of Illinois Chicago (UIC). The dataset comprised two-dimensional near-infrared (NIR) scanning laser ophthalmoscopy (SLO)-based fundus images and three-dimensional OCT images acquired using the Heidelberg Spectralis (Heidelberg Engineering, Heidelberg, Germany) imaging system. A total of 531 eyes were included: 79 eyes from 49 healthy control subjects, 119 eyes from 88 patients with DR without DME, and 333 eyes from 174 patients diagnosed with DME. Demographic characteristics of the study population are summarized in Table 1. Among the DME eyes, 123 were identified as having ci-DME. All subjects were evaluated and clinically diagnosed at the UIC Eye Clinic. All patients with diabetes had Type 2 Diabetes Mellitus. Patients who had received any prior ocular treatment before presentation were excluded. Additional exclusion criteria included the presence of epiretinal membrane and macular edema due to causes other than DME. Image quality screening was performed during preprocessing, and images with poor quality were excluded from the analysis.

Each input fundus image was preprocessed to isolate the macular region. The macular area was cropped based on the OCT scan localization displayed on the corresponding SLO fundus image and then resized to a fixed resolution of 512 × 384 pixels to standardize the input dimensions for the DL model. The preprocessing ensured uniform spatial coverage of the macula across all input images. To generate ground truth for training, corresponding OCT volume profiles were exported for each eye. Retinal thickness was defined for each A-scan as the distance between the inner limiting membrane (ILM) and Bruch’s membrane (BM), as segmented by the Heidelberg software (version 1.10.4.0). The OCT scans and corresponding automated segmentations were reviewed for quality, and scans with segmentation errors or poor image quality were excluded from the dataset. The thickness values across the B-scans were interpolated to create a dense, continuous, pixel-wise retinal thickness map covering the entire macular region. This thickness map was then spatially aligned to the corresponding fundus image using the Heidelberg device–provided OCT–SLO co-registration and resized to match the 512 × 384 resolution of the preprocessed input fundus images. The thickness values were first normalized to a range of 0–255 to ensure consistent correspondence between input and ground truth. Subsequently, all thickness maps were min–max normalized to the range [0, 1], which served as the final input scale for model training.

**Figure 1:**
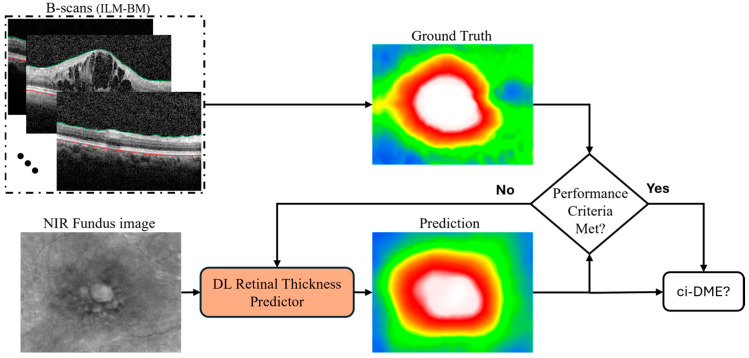
Representative pipeline showing input fundus image and OCT-derived ground truth for DL-based retinal thickness prediction and ci-DME detection.

The resulting paired dataset was used to train a DL model. During training, the model learned to predict the pixel-wise thickness of the retina from input using a customized loss function that evaluated discrepancies between predictions and the OCT-derived ground truth. As illustrated in Figure 1, this process enabled the model to learn a direct mapping from fundus images to retinal thickness distribution. In the inference phase, the trained model takes a fundus image as input and outputs a corresponding pixel-wise retinal thickness map. This predicted map can be used to detect the presence of ci-DME and assess its severity.

### 2.2. DL and Implementation Procedures

**Figure 2:**
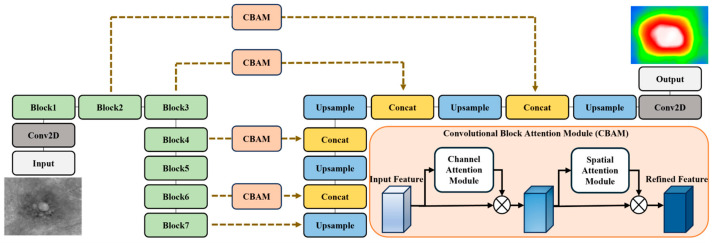
Representative DL model utilizing input fundus image and predicting retinal thickness.

All input fundus images were min–max normalized to the range [0, 1] prior to model training. A DL model (Figure 2) was employed to predict pixel-wise retinal thickness from 2D fundus images using a U-Net–based architecture [32], integrated with Convolutional Block Attention Modules (CBAM) [33] in the skip connections. The encoder used EfficientNetB0 [34] as its backbone for feature extraction. The decoder progressively reconstructed full-resolution retinal thickness maps through upsampling and feature concatenation.

Transfer learning was employed to address the challenge of limited dataset size [35] in the fundus image dataset. Pretrained weights from the ImageNet dataset were loaded into the EfficientNet-B0 encoder, allowing the network to benefit from general features learned from large-scale images [36]. The model was subsequently fine-tuned for the pixel-wise task of retinal thickness prediction. To mitigate the risk of overfitting and enhance the model’s generalizability, several data augmentation techniques were applied during training. These included random rotations (±20°), horizontal flipping, brightness adjustment (0.5–1.7), random zooming (0.8–1.2).

Training was conducted for up to 600 epochs with an initial learning rate of 0.0001. A dynamic learning rate scheduler was used to reduce the learning rate by a factor of 0.5 when validation loss plateaued for 10 consecutive epochs. Optimization was performed using the Adam optimizer. The loss function was defined as a weighted combination of mean squared error (MSE) and structural similarity index measure (SSIM), where the final loss was computed as:

(1) $$L=\lambda \cdot MSE+\left(1-\lambda \right)\cdot \left(1-\;SSIM\right)$$

where λ was set to 0.4 based on validation performance to balance MSE and SSIM, and a batch size of 4 was used throughout training. To safeguard against overfitting and ensure that the best-performing model was retained, a checkpointing strategy was applied to save the model weights corresponding to the lowest validation loss. A five-fold cross-validation strategy was implemented, with data partitioned at the patient level to ensure that images from the same participant, including both eyes, were assigned to the same fold and to prevent data leakage. In each fold, 80% of patients were used for training and the remaining 20% for validation, ensuring that every image served as part of the validation set exactly once across the five folds. Performance metrics were averaged across all folds to provide a robust assessment of the model’s accuracy and generalizability. The entire pipeline was implemented in Python 3.8 using Keras 2.9.0 with TensorFlow 2.9.1 as the backend. Model training was conducted on a Windows 10 workstation equipped with an NVIDIA Quadro RTX 6000 GPU.

### 2.3. Performance Evaluation

The performance of the proposed DL model was evaluated using mean absolute error (MAE), root mean squared error (RMSE), and SSIM. MAE quantified the average absolute difference between the predicted and ground truth retinal thickness values at the pixel level. RMSE emphasized larger prediction errors by computing the square root of the average squared pixel-wise differences, providing sensitivity to outliers. SSIM, in contrast, evaluated the overall structural similarity between predicted and reference thickness maps across the full macular region. To assess regional prediction accuracy, we calculated the MAE between the model-predicted and OCT-derived retinal thickness values within each of the nine subfields defined by the Early Treatment Diabetic Retinopathy Study (ETDRS) grid. This subfield-based comparison allowed for evaluation of the anatomical and clinical accuracy of the model’s predictions across the macular region. To assess the model’s ability to identify clinically significant DME, we conducted a secondary classification analysis using a central subfield thickness (CST) threshold of ≥300 μm to differentiate ci-DME from non-ci-DME eyes. This threshold is consistent with criteria used in prior clinical studies evaluating ci-DME [37]. Classification performance was evaluated using accuracy, sensitivity, specificity, positive predictive value (PPV), negative predictive value (NPV), and the area under the receiver operating characteristic curve (AUROC).

## 3. Results

**Figure 3:**
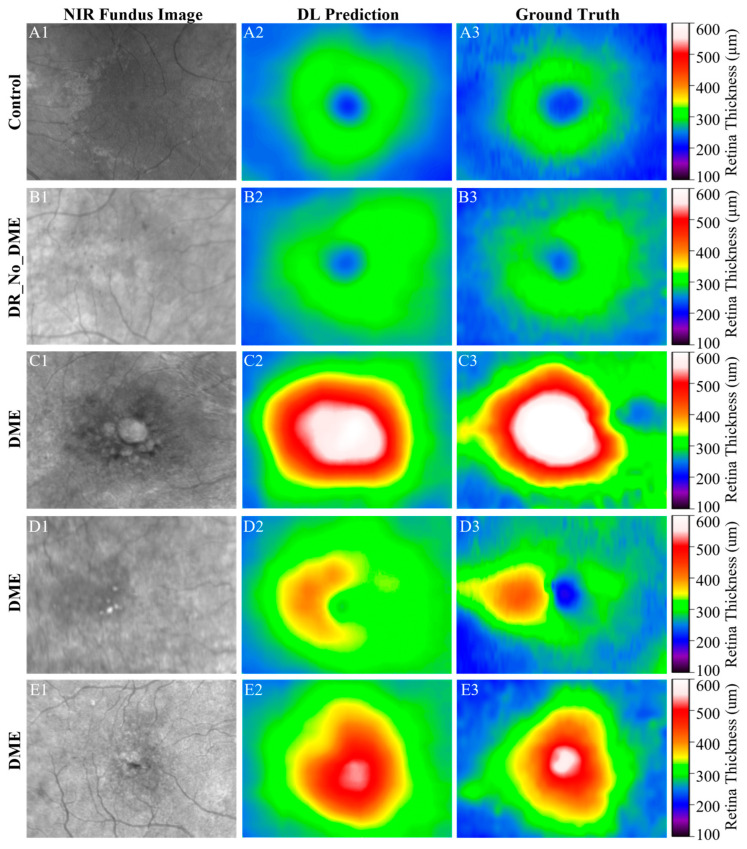
Representative qualitative comparison of DL-prediction and OCT-derived ground truth retinal thickness. Each row shows an input fundus image (**left**), DL-prediction (**middle**), and OCT-derived thickness map (**right**) for a control (**A1**–**A3**), DR without DME (**B1**–**B3**), and DME cases (**C1**–**C3**,**D1**–**D3**,**E1**–**E3**).

Figure 3 presents a comparison between the DL–predicted retinal thickness maps and the OCT-derived ground truth. Representative examples include a control subject (Figure 3(A1–A3)), a patient with DR without DME (Figure 3(B1–B3)), and three patients with DME (Figure 3(C1–C3,D1–D3,E1–E3)). In the control and DR without DME cases, the predicted thickness maps (Figure 3(A2,B2)) closely align with the ground truth (Figure 3(A3,B3)), particularly in the foveal region, an area critical for high-acuity vision. For the DME cases, the model accurately detected regions of retinal thickening. The predictions (Figure 3(C2,D2,E2)) show strong structural similarity to the ground truth (Figure 3(C3,D3,E3)).

The model demonstrated strong predictive performance across groups, capturing spatial retinal thickness patterns consistent with established anatomical and physiological characteristics. These findings indicate that the DL model preserves global retinal structure and accurately reflects disease-related changes in retinal morphology. In DME cases, the model further identified macular thickening associated with fluid accumulation and hard exudates, highlighting its ability to localize pathological alterations.

**Figure 4:**
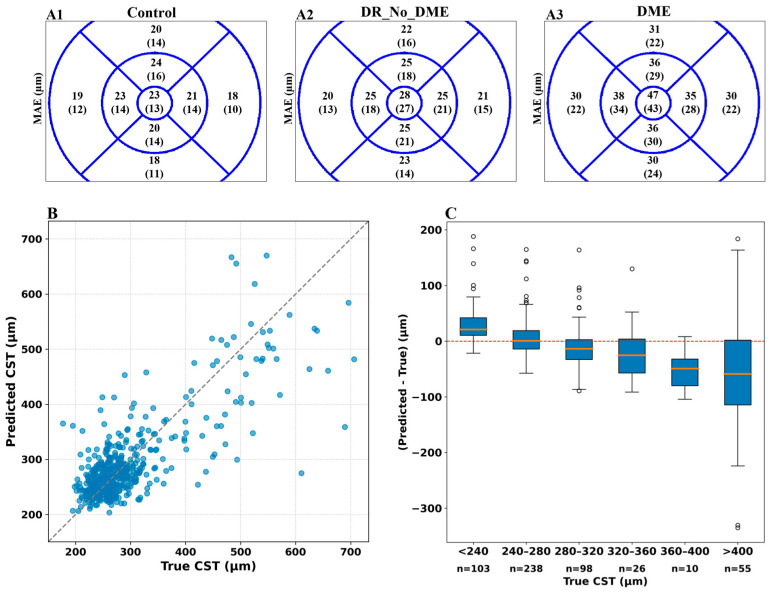
Representative quantitative comparison of DL-prediction and OCT-derived ground truth retinal thickness. (**A1**–**A3**) MAE between DL-prediction and ground truth values across ETDRS subfields for a control (**A1**), DR without DME (**A2**), and DME cases (**A3**). (**B**) Scatter plot showing the comparison between predicted and true CST values with the identity line indicated. (**C**) Prediction errors stratified by true CST ranges.

Quantitative analysis of model performance showed an MAE of 30.41 ± 18.68 µm, an RMSE of 36.14 ± 21.05 µm, and an SSIM of 0.87 ± 0.04 across the entire macular region. To further assess regional performance, we computed the MAE between the DL-prediction and OCT-derived ground truth within each ETDRS subfield. Figure 4(A1–A3) illustrate the distribution of MAE values across ETDRS subfields for all three groups. The model exhibited the lowest errors in the control group, with slightly higher but still acceptable deviations in the DR without DME group. In the DME group, although the MAE was elevated due to increased structural variability, the model maintained reasonable agreement with ground truth values, demonstrating its applicability across a range of disease severities.

Comparison of predicted and ground truth CST values was performed using a scatter plot (Figure 4B) and stratified error analysis (Figure 4C). The scatter plot shows that most predicted CST values cluster closely around the identity line, indicating strong agreement for the majority of cases. However, points become more widely dispersed at higher CST values, with a visible tendency toward underestimation in eyes with greater retinal thickness. Stratified error analysis further quantifies this trend, showing that eyes with CST values below 360 µm had lower absolute prediction errors, whereas eyes above this threshold demonstrated larger negative errors, with a MAE of 93.08 ± 66.95 µm. This pattern highlights the increased difficulty of accurately estimating severe macular edema.

**Figure 5:**
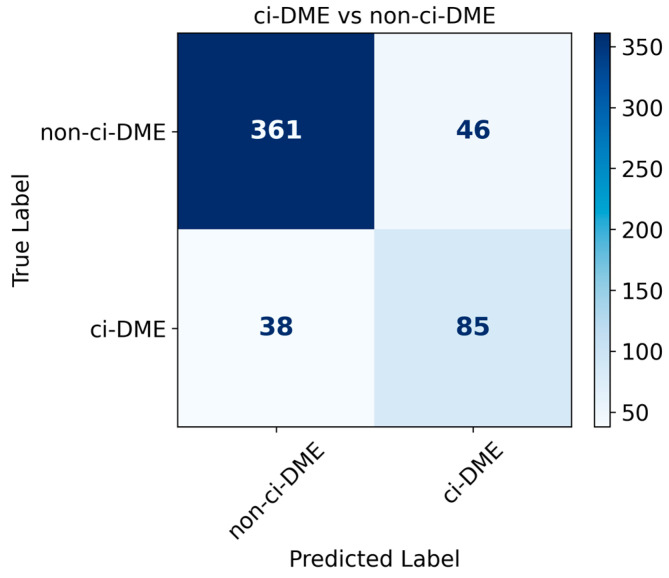
Confusion matrix for ci-DME classification.

To evaluate the clinical relevance of the predicted thickness maps, we performed a classification of ci-DME versus non-ci-DME cases using a CST threshold of 300 µm, as predefined in Section 2. The model achieved AUROC of 0.869 (95% CI, 0.826–0.906), with an accuracy of 84.1% (95% CI, 80.60–86.84), sensitivity of 69.1% (95% CI, 60.04–76.06), specificity of 88.7% (95% CI, 85.22–91.40), PPV of 64.8% (95% CI, 56.39–72.53), and NPV of 90.5% (95% CI, 87.05–92.92). The corresponding confusion matrix is shown in Figure 5, where 85 of 123 ci-DME cases were correctly classified, while 38 were misclassified as non-ci-DME. Conversely, among 407 non-ci-DME cases, 361 were correctly classified, with 46 false positives.

To further evaluate robustness, a sensitivity analysis was performed using alternative CST thresholds of 280 µm and 320 µm. At 280 µm, the model demonstrated increased sensitivity of 79.84% (95% CI, 71.93–85.95) with reduced specificity of 73.65% (95% CI, 69.15–77.69). At 320 µm, sensitivity decreased to 58.06% (95% CI, 49.26–66.38) with increased specificity of 94.09% (95% CI, 91.36–95.99). Overall, these results demonstrate a consistent threshold-dependent trade-off between sensitivity and specificity with higher sensitivity achieved at lower thresholds and higher specificity at higher thresholds.

**Figure 6:**
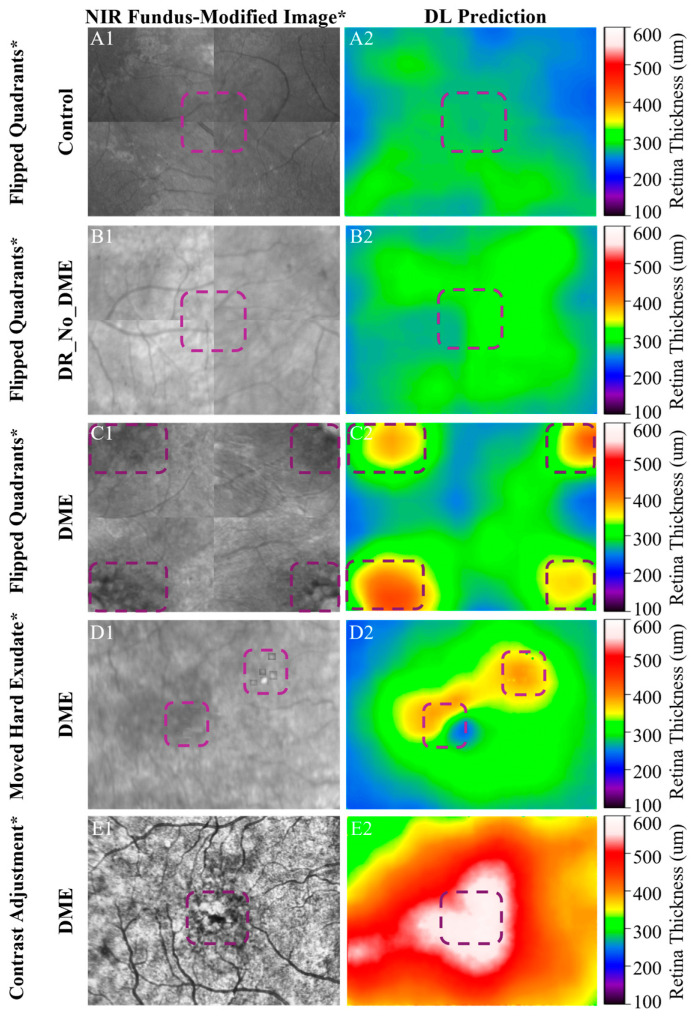
Comparative analysis of the effect of fundus features on the model performance for retinal thickness prediction. Each row shows a modified input fundus image (**left**) and the corresponding DL-prediction (**right**). (**A1**,**A2**,**B1**,**B2**) Flipping image quadrants to disrupt the vascular pattern. (**C1**,**C2**) Shifting pathological features to peripheral regions. (**D1**,**D2**) Relocating hard exudates. (**E1**,**E2**) Enhancing image contrast using CLAHE.

To further investigate the mechanisms underlying the model’s predictions, we performed controlled input manipulation experiments. In one setting, fundus images were divided into quadrants and spatially flipped, disrupting the native anatomical arrangement. As shown in Figure 6(A1,B1), this manipulation altered the spatial organization of superficial vascular structures surrounding the foveal region. The corresponding predictions (Figure 6(A2,B2)) failed to correctly localize the fovea when compared with the ground truth thickness maps (Figure 3(A3,B3)), suggesting an association between vascular spatial patterns and positional encoding. This observation is consistent with known retinal anatomy: the fovea is a specialized central depression characterized by the foveal avascular zone, where retinal capillaries are absent, and is surrounded by a network of superficial vessels. These vascular patterns provide a stable anatomical reference frame that can be used to infer retinal location and guide spatially consistent thickness estimation. The results therefore suggest that vascular spatial configuration may contribute to spatial localization of predictions, rather than relying solely on local intensity features. Disruption of this global vascular context was associated with prediction mislocalization, providing qualitative evidence that spatial coherence may influence the learned representation.

In another experiment, the abnormalities in the original input fundus image (Figure 3(C1)) were shifted toward the image periphery using the same quadrant-flipping technique (Figure 6(C1)), thereby disrupting their normal anatomical context. Despite this relocation, the model continued to predict increased retinal thickness in these regions (Figure 6(C2)), even though such spatial configurations are clinically atypical. This qualitative observation suggests that the model may be sensitive to local image features associated with pathology, including intensity irregularities and textural disruptions, rather than relying solely on anatomically consistent spatial priors. In other words, the presence of pathology-like visual patterns appears sufficient to trigger predictions of increased thickness, even when those patterns are presented in implausible locations, suggesting a potential dependence on local appearance-based cues.

We further examined the model’s sensitivity to clinically relevant biomarkers, particularly hard exudates, which are well-established indicators of vascular leakage and lipid deposition in DME. In one illustrative case, the original input fundus image (Figure 3(D1)) was modified by removing hard exudates and reinserting them into alternative retinal locations (Figure 6(D1)). The model consistently predicted localized retinal thickening at these new positions (Figure 6(D2)), suggesting that the presence and spatial context of hard exudate-related visual patterns may be associated with changes in predicted thickness. These findings should be interpreted as qualitative and hypothesis-generating rather than as direct evidence of the features driving model decisions, and are consistent with clinical understanding, as hard exudates are often found in regions of chronic leakage and are spatially associated with areas of retinal thickening, although they are not a direct measure of thickness themselves. The model’s response is consistent with the possibility that it has learned associations between these lesions and underlying fluid accumulation, potentially using them as indirect markers of disease activity.

Finally, we enhanced the contrast of the original input fundus image (Figure 3(E1)) using Contrast-Limited Adaptive Histogram Equalization (CLAHE) [38]. A comparison between the prediction from the original input image (Figure 3(E2)) and the prediction from the contrast-enhanced input image (Figure 6(E2)) revealed a visual correspondence between regions of local contrast variation and predicted retinal thickening. This observation suggests that the model is sensitive to contrast variations that may reflect underlying structural changes in the retina, rather than directly interpreting contrast as a standalone indicator of edema. In NIR imaging, DME is often associated with hyporeflective regions corresponding to intraretinal fluid accumulation, sometimes accompanied by surrounding hyperreflective boundaries due to changes in light scattering at fluid–tissue interfaces [39], thereby producing localized contrast variations. These contrast patterns may serve as indirect cues that the model has learned to associate with retinal thickening. Accordingly, these qualitative experiments suggest that contrast-based features, together with other structural and contextual information, may contribute to the model’s predictions of edema-related changes.

## 4. Discussion

In this study, we demonstrated that pixel-wise retinal thickness maps can be reliably predicted from NIR fundus images using a DL model, achieving an MAE of 30.41 ± 18.68 µm and an SSIM of 0.87 ± 0.04 across the macula, with consistent performance across ETDRS subfields. The model also enabled ci-DME classification with 84.1% accuracy, 69.1% sensitivity, and 88.7% specificity. Interpretability analyses further highlighted vascular architecture, hard exudates, and local contrast variations as the principal image-level surrogate markers through which NIR fundus images encode retinal thickness information.

The strong predictive performance is grounded in the physical properties of NIR reflectance imaging that distinguish it from conventional color fundus photography. Because NIR light penetrates deeply into ocular tissues, reaching the RPE and choroid, and the resulting two-dimensional fundus image integrates optical signals from multiple retinal layers simultaneously. Structural changes associated with DME, such as intraretinal fluid accumulation, alter tissue scattering and reflectance properties at depth, producing measurable contrast signatures in the NIR image even without depth-resolved scanning. This depth-integrating character provides a richer substrate for thickness estimation than visible-light imaging, which is dominated by superficial retinal reflectance. The relatively high SSIM reflects not only pixel-level accuracy but spatial coherence; the model preserved the characteristic foveal depression and parafoveal thickness gradient, which are clinically critical landmarks. The observed underestimation in high-CST cases is consistent with the known difficulty of inferring large fluid volumes from surface reflectance alone, and with the limited representation of severe edema in the training cohort; this is a known challenge across modality-translation tasks and is addressable through dataset expansion.

Interpretability analyses provide insight into how the model decodes thickness information from NIR images, and why this is physically plausible. The dependence on vascular spatial patterns for foveal localization reflects the well-established anatomical relationship between the foveal avascular zone and the surrounding capillary network, a stable, imaging-modality-independent landmark that the model learned to use as a spatial reference frame for thickness map registration. The sensitivity to hard exudates is consistent with their known co-localization with chronic vascular leakage and retinal thickening in DME; although exudates are not direct measures of thickness, they are reliable spatial indicators of underlying fluid accumulation that the model has learned to leverage. The response to local contrast variations is consistent with visual patterns observed in NIR fundus images of intraretinal fluid regions, where hyporeflective areas and surrounding intensity transitions contribute to local contrast differences. These observations suggest that the model leverages informative image-level patterns for retinal thickness prediction, as revealed by qualitative interpretability analyses.

Previous studies have primarily focused on detecting ci-DME from fundus photographs, relying solely on binary classification. For instance, Varadarajan et al. [25] and Liu et al. [40] developed DL models to identify ci-DME, demonstrating comparable sensitivity and higher specificity relative to human experts. Tan et al. [41] further emphasized the clinical utility of AI tools in detecting ci-DME with visual impairment, highlighting their potential role in prioritizing treatment. In contrast, our model not only performs ci-DME classification but also generates continuous, full-field, pixel-level retinal thickness maps from NIR fundus images. The predicted maps provide continuous thickness values across the entire macula, capturing regional variability in retinal structure. Such granularity supports both qualitative interpretation and quantitative assessment of disease severity. Although the proposed framework achieved a specificity of 88.7% for ci-DME classification, its sensitivity of 69.1% indicates that some cases of center-involved disease may not be detected. Therefore, the model may be considered as a risk-support tool for screening-related analysis of ci-DME features, to complement, rather than replace, comprehensive ophthalmic evaluation. In decentralized settings, individuals identified as high risk could potentially be referred for further ophthalmic evaluation and confirmatory OCT imaging, while future work may further optimize the sensitivity-specificity trade-off. The generation of continuous, full-field retinal thickness maps is particularly useful for longitudinal analysis of retinal structure, where accurate assessment of disease progression is important for guiding clinical evaluation.

Several limitations should be noted. The model exhibited increased underestimation errors in eyes with high CST values. In particular, eyes with CST values exceeding 360 µm demonstrated a higher MAE (93.08 ± 66.95 µm), highlighting the greater difficulty of accurately estimating severe macular edema. This limitation likely reflects both the inherent challenge of inferring large fluid volumes from two-dimensional NIR reflectance images and the limited representation of severe edema cases in the training dataset. Future work should prioritize expanding the number of high-CST cases to improve performance in this clinically important subgroup. More broadly, the training data were acquired using a single imaging device and a specific NIR-SLO protocol, which may limit robustness when applied to images from other devices with differing optical properties or acquisition parameters. Such domain shifts could reduce performance in real-world deployment, and external validation is required to assess generalizability and clinical feasibility. Future work should therefore focus on multi-center, multi-device validation and prospective evaluation to establish robustness and feasibility of the approach under real-world condition.

## 5. Conclusions

This study demonstrates that NIR fundus imaging, leveraging its nonmydriatic acquisition and depth-integrating optical properties, may serve as a practical substrate for DL-based retinal thickness estimation. The predicted pixel-wise thickness maps showed strong agreement with OCT-derived ground truth and supported accurate ci-DME classification, suggesting potential applications in screening, triage support, and treatment-related assessment that warrant further external and prospective validation. Interpretability analyses were performed to qualitatively visualize image regions contributing to model predictions. These visualizations highlighted vascular architecture, hard exudates, and local contrast variations as image-level features associated with model predictions. Together, these results suggest that NIR fundus imaging may serve as a scalable and cost-effective imaging modality for DME monitoring, with potential applications in longitudinal assessment and treatment-response evaluation that require further validation, particularly in resource-limited settings where OCT is not readily available.

## Figures and Tables

**Table 1:** Demographics of Subjects.

Metric	Control	DR No DME	DME
No. of Subjects	49	88	174
Male	26	40	92
Female	23	48	82
Age (mean ± SD)	56.26 ± 13.17	59.31 ± 11.12	59.48 ± 13.04
Age range	26–72	27–78	25–91
No. of Images	79	119	333
Right Eye	38	66	185
Left Eye	41	53	148

## Data Availability

The data supporting the conclusions of this article will be made available by the authors on request. The trained model weights will also be made available upon reasonable request.

## Abbreviations

The following abbreviations are used in this manuscript:

OCT	Optical coherence tomography
DME	Diabetic macular edema
NIR	near-infrared
SLO	scanning laser ophthalmoscopy
DR	Diabetic retinopathy
DL	Deep Learning
